# Study on rheological properties of POE/VO compound recycled asphalt

**DOI:** 10.1371/journal.pone.0293648

**Published:** 2023-11-09

**Authors:** Hua Mei, Zhaoxia Hu, Zhenyuan Zheng, Junyi Zeng, Lei Yu

**Affiliations:** 1 School of Civil and Architecture Engineering, Hunan Institute of Technology, Hengyang, P. R. China; 2 College of Urban and Rural Construction, Zhongkai University of Agriculture and Engineering, Guangzhou, P. R. China; Shandong University of Technology, CHINA

## Abstract

In order to improve the high temperature performance of Vegetable oil recycled asphalt, this study used Polyolefin elastomer (POE) and vegetable oil (VO) to compound recycled aging asphalt. The properties of recycled asphalt were compared and analyzed by conventional physical properties and high & low temperature rheological tests. The results show that 8% VO content can achieve the best regeneration effect. Based on this VO dosage, a variety of POE/VO combination mixture schemes were designed and tested to obtain excellent deformation resistance of recycled aging asphalt under high temperature environments. The POE/VO combination with an appropriate dosage can restore the high temperature deformation resistance and elastic recovery performance even beyond the pre-aging level, and increase the critical temperature by 4~10°C. Considering the physical properties and rheological properties of asphalt, the recommended ratio of POE/VO composite recycled asphalt is 8% VO+4% POE and 8% VO+6% POE.

## 1. Introduction

With the continuous development of highway construction in China, a large amount of asphalt raw materials is consumed in the construction and maintenance of roads every year, and a large amount of waste asphalt mixture is produced in the maintenance of old roads. If this situation cannot be effectively dealt with, it will face an increasingly serious shortage of asphalt pavement raw materials and a large amount of waste asphalt mixture accumulation [1–3]. Therefore, the recycling of aged asphalt mixture through regenerant is the best solution, which can greatly reduce the consumption of petroleum asphalt and reduce the accumulation of aged asphalt material, and has significant economic and social benefits, it is an important research question of asphalt pavement maintenance of recycling asphalt [4–6].

The key problem of recovery and utilization of aged asphalt material is to restore the physical and rheological properties of aged asphalt [6]. Rejuvenators is a kind of low viscosity substance, which can repair the physical and rheological properties of aging asphalt [6]. The main component of the traditional asphalt rejuvenators is mineral oil, which are widely used to restore the aged asphalt performance, but there are still many technical problems [7, 8]: the light components of it rejuvenators are easily volatilized at high temperature, so the regeneration efficiency of it rejuvenators is relatively poor; the content of aromatics and unsaturated bonds of mineral oil rejuvenators is relatively high; thus, rejuvenators are oxidized easily at high temperature. The aging resistance and durability of mineral oil rejuvenators are reduced. In addition, its composition of polycyclic aromatic hydrocarbons (PAHs) may cause human health and environmental hazards [9, 10]. Meanwhile, the cost of mineral oil rejuvenators as a petroleum product is also high, which consumes non-renewable resources [11]. Therefore, the economic benefits of recycled pavement are not significant.

In recent years, it has been found through research that vegetable oil (VO) is rich in unsaturated fatty acid esters and light oil components, and has the characteristics of low volatility, low toxicity and environmental protection. VO can supplement the light oil components lost in aging asphalt, and realize the synergistic effect of various components to restore the rheological properties of asphalt [12–14]. Compared with mineral oil, vegetable oil has a high boiling point and is not easy to lose during construction [15]. Therefore, the use of vegetable oil for asphalt pavement regeneration has been favored by road researchers, researchers have developed soybean oil [16], cashew shell oil [17], peanut oil [18] and other vegetable oil regeneration agents. However, the high temperature performance of asphalt reclaimed by vegetable oil regenerator still needs to be further improved [19]. Existing scholars have combined rubber [20], plasticizer [21], anti-aging agent [22] and other auxiliary additives with vegetable oil to prepare a composite regenerant for asphalt regeneration, in order to modify asphalt on the basis of asphalt regeneration, so as to fully restore the performance of aging asphalt.

Polyolefin elastomer (POE) is an ethylene-octene copolymer synthesized by a catalyst with limited geometry. The molecular of POE has a narrow molecular weight distribution, a short-branched chain structure, and not contain unsaturated bonds [23], which yields it the advantages of high elasticity and high elongation. POE has a good toughness, mechanical properties, heat resistance and processing properties [24, 25]. By adding POE to SBS, the aging resistance and high temperature resistance of POE/SBS composite modified asphalt have been improved [23, 26, 27]. Ran [25] studied POE graft copolymer as matrix asphalt modifier, harvested this result what the modifier is arranged in a network of sheets in the bitumen phase, the high temperature performance of asphalt is improved, also the temperature reduced and the PG grading improved. Ye [28] studied the results show that polyolefin has two glass transition points which facilitate the simultaneous improvement of the high and low-temperature properties of asphalt.

In conclusion, VO can repair the physical and rheological properties of aged asphalt, but it simultaneously reduces the high-temperature rutting resistance, whereas POE can improve the high-temperature performance of asphalt. Currently, researchers mainly pay attention to the application of VO on the rejuvenating aged asphalt. However, there are few studies on modification of VO rejuvenated recycled asphalt. In this paper, the change rule of rheological properties of recycled asphalt mortar will be compared and analyzed. Based on the blend complex regeneration technology, considering three indexes, Rotation Viscosity (RV), Frequency Sweep (FrS) and Temperature Sweep (TeS), the multi-stress creep recovery test (MSCR) and low temperature bending beam rheology test (BBR) will be conducted. The best ratio of POE/VO compound recycled asphalt and the mechanism of compound recycled asphalt can be determined. This study will provide feasible methods and technical support for environmentally friendly modification of recycled asphalt.

## 2. Materials and testing methods

### 2.1 Materials

In this study, SK-90# road petroleum asphalt was selected as the base asphalt, and aged asphalt was obtained by the aging process of RTFOT (technical indexes as shown in Table 1). Vegetable oil was selected as the fresh peanut oil purchased in the market (technical indexes as shown in Table 2). The thermoplastic elastomer was selected as heat-resistant and anti-aging POE-8203 (technical indexes as shown in Table 3). Sulfur was selected as the crosslinking agent.

**Table 1:** Technical indicators of 90# asphalt.

Index	25°C Penetration (0.1mm)	Softening Point (°C)	10°C Elongation (cm)	135°C Rotary Viscosity (mPa∙s)	RTFOT		
					Quality Loss (%)	Residual Needle Penetration Ratio (25°C) (%)	Residual Elongation (15°C) (cm)
Value	93.9	46.8	54.0	369.0	-0.18	93.0	>100

**Table 2:** Technical indicators of vegetable oil.

Index	Colour	Density (g/cm^3^) (20°C)	TG Starting Decomposition Temperation	Flashing Point (°C)	60 Viscosity (Pa∙s)
Value	Yellow	0.910	280	312	0.160

**Table 3:** Technical indicators of POE-8203.

Index	Density (g/cm^3^)	Tensile Stress (MPa)	Tensile Strain (%)	Flexural Modulus (MPa)	Brittle Temperature (°C)	Melting Temperature (°C)
Value	0.882	22	820	24	<-76.0	74

### 2.2 Preparation method of recycled asphalt

**Fig 1:**
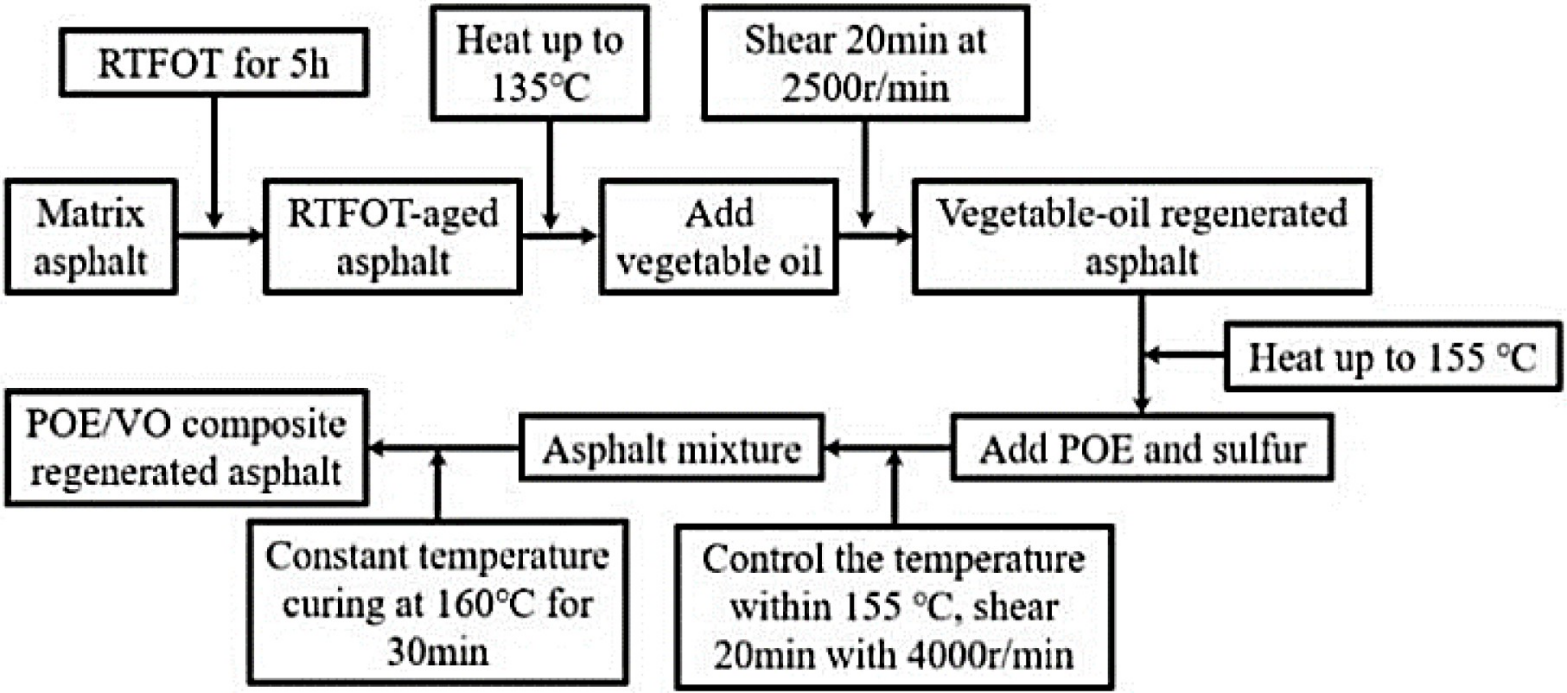
Preparation process of POE/VO reclaimed asphalt.

Fig 1 shows the specific preparation process of recycled asphalt, details as follows: (1) The first step is to prepare aged asphalt refer to ASTM D2872-12 [29], the matrix asphalt is used for RTFOT aging at 163±1°C for 5h. (2) VO recycled asphalt is prepared, when the temperature of the oil bath heating system is controlled about 135°C, the dosage of VO (2wt.%, 4wt.%, 6wt.%, 8wt.% and 10wt.%) is added to the aged asphalt, and the ZD-300D high-speed shear is used to shear the asphalt for 20min at 2500r/min. (3) After the oil bath system was heated up to 155°C, VO recycled asphalt are mixed with the dosage of 0.1wt.% sulfur and different POE (2wt.%, 4wt.%, 6wt.% and 8wt.%). After such above steps finished, it should be sealed in a 160°C constant temperature oven for swelling and development for 30 min, and set aside for more than 10h, finally, POE/VO compound recycled asphalt is prepared.

### 2.3 Testing methods

#### Fundamental physical properties

These asphalt indexes of 25°C penetration degree, softening point and 15°C ductility index are mainly conducted according to ASTM D5 [30], ASTM D 36 [31] and ASTM D113 [32]. The specific test of RTFOT aging separately referred to ASTM D2872-12 [29].

#### High-temperature viscosity test

The viscosity of asphalt is used to indicate the viscosity of asphalt, which refers to the ability of asphalt material to resist shear deformation under external force. The 135°C viscosity of asphalt was determined by Brockfield viscometer according to ASTM D4402 [33] test.

#### Dynamic shear rheometer test

DSR is selected considering rutting property. The specific test method referred to AASHTO T 315 [34]. DSR test is mainly conducted by Anton Parr SmartPave-102 DSR instrument with 25mm rotor and 1mm parallel plate spacing to test the complex shear modulus (G*) and phase angle (*δ*). FrS test is conducted in a controlled strain mode with a scanning range of 0.1–10 Hz and a temperature range of 16~64°C at intervals of 12°C. TeS test is carried out with frequency of 10rad/s and temperature range of 20°C~ 80°C. Multiple stress creep recovery (MSCR) test is carried out in loading and unloading cycles at 58°C, 0.1kPa and 3.2kPa stress levels, and the mean recovery rate *R(τ)* and the differential ratio *Jnr*_*diff*_ between non-recoverable creep compliance *Jnr(τ)* and non-recoverable creep compliance is determined (as shown in Eqs 1–3).

$$R=\frac{\gamma_{m}-\gamma_{r}}{\gamma_{m}-\gamma_{0}}$$ (1)

$$J_{nr}=\frac{\gamma_{r}-\gamma_{0}}{\tau }$$ (2)

$$J_{nr-diff}=\frac{J_{nr(3.2)}-J_{nr(0.1)}}{J_{nr(0.1)}}\times 100$$ (3)

Where, *γ*_*0*_ is the cyclic shear strain at the beginning; *γ*_*m*_ is the cyclic peak strain; *γ*_*r*_ is the cyclic unrecoverable strain; *τ* is the loading stress.

#### Bending beam rheometer test

BBR is selected considering cracking property. Refer to the method of ASTM D 6648 [35]. The creep stiffness modulus (*S*_*m*_) and creep rate (*m*) of recycled asphalt is tested by cannon TE-BBR instrument at −12°C、−18°C and −24°C respectively.

## 3. Rheological property of reclaimed asphalt

### 3.1 Three indexes and RV analysis

**Fig 2:**
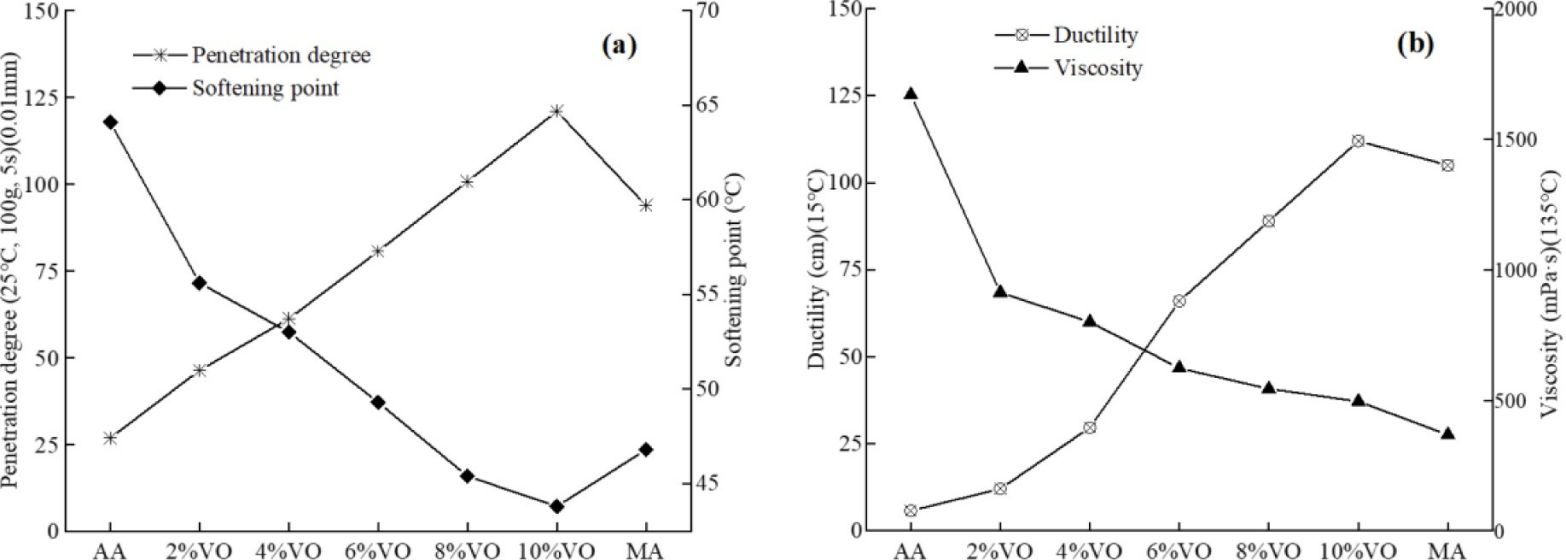
The four indexes change of recycled asphalt with different VO. (a) penetration and softening; (b) ductility and viscosity. AA denotes aging asphalt; MA denotes matrix asphalt.

Fig 2 shows the test results of three indexes and viscosity of different reclaimed asphalt. It can be found that compared with the matrix asphalt, the penetration and elongation of the aging asphalt are reduced, while the softening point and viscosity are increased. It is because that the thermal oxygen aging reduces the content of light and oily components in the asphalt mortar, such as aromatic compounds, resulting in an increase in the stiffness of the mortar [36]. With the VO content increasing from 2% to 10%, the penetration degree of VO recycled asphalt mortar gradually increases, and is higher than that of base asphalt at 10% VO content, that is, VO can soften the aging asphalt and reduce the consistency and viscosity of the mortar. The softening point decreased gradually, and it of 8% and 10% VO content is lower than the level before aging, but it is not good for the high temperature performance of asphalt mortar that softening point display too low. The ductility shows a gradual upward trend, and exceeds the pre-aging level at 10% content, it indicates that VO addition can significantly improve and restore the low-temperature. The viscosity decreases gradually with the increase of VO content, that is, VO reduces the ability of the mortar to resist shear and flow deformation under external force. According to the above analysis about the properties of VO recycled asphalt, combined with the construction regulation, the optimal VO content is 8%. However, compared with the pre-aging, it is still a large gap in needle penetration, softening point and viscosity of 8% VO recycled asphalt.

**Fig 3:**
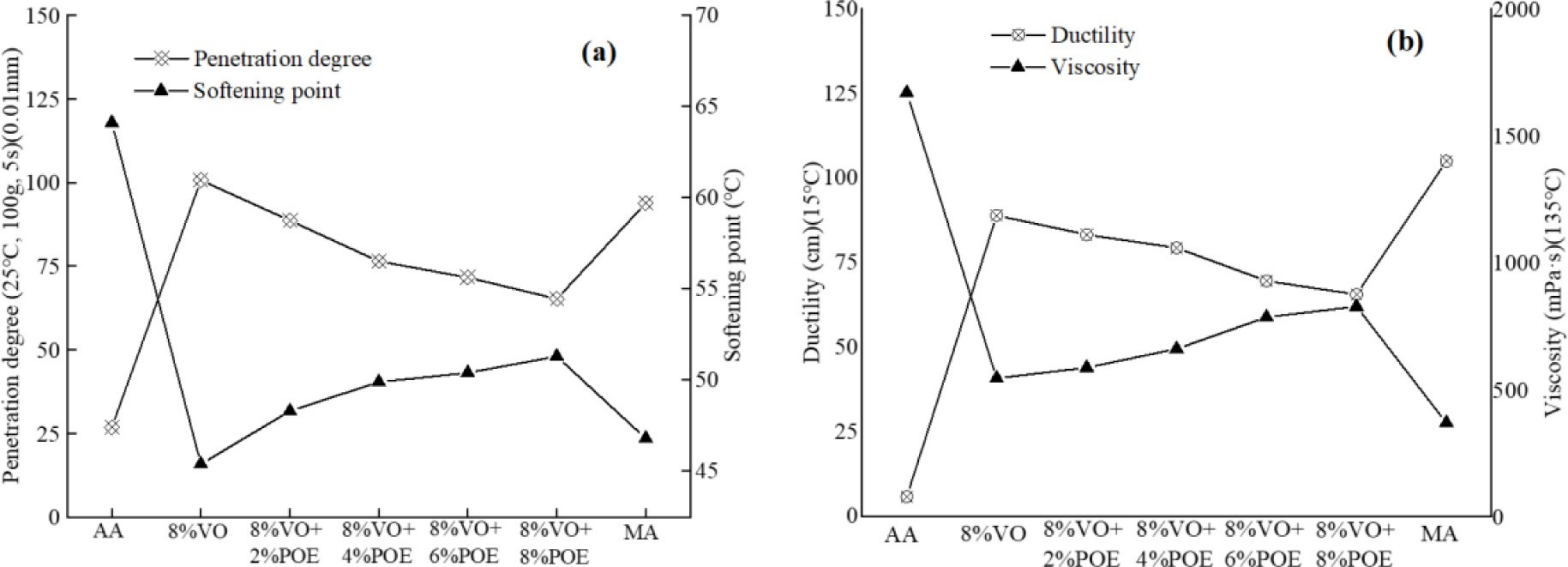
The four indexes change of recycled asphalt with different VO+POE. (a) penetration and softening; (b) ductility and viscosity. AA denotes aging asphalt; MA denotes matrix asphalt.

Fig 3 shows the three major indexes and viscosity test results of POE/VO composite recycled asphalt. According to the figure, the addition of POE can thicken and harden the VO recycled asphalt, that is, the penetration of recycled asphalt mortar decreases, and the softening point increases and the viscosity becomes larger with the increase of POE content. For example, compared with pure VO recycled asphalt, the penetration rate of the asphalt is respectively reduced by 12.02% (VO+2%POE), 23.13% (VO+4%POE), 28.89% (VO+6%POE) and 35.25% (VO+8%POE), but softening point respectively increases by 6.39% (VO+2%POE), 9.91% (VO+4%POE), 11.01% (VO+6%POE) and 12.99% (VO+8%POE). It shows that the addition of POE can improve the high temperature performance of VO recycled asphalt, the main reason may be that POE elastoids absorb the light oily components in asphalt mortar and produce volume expansion, while the octene chain structure and crystalline ethylene chain form many physical links, which limit the mobility of asphalt mortar [27, 37]. In addition, it is not enough to fully analyze and judge the ductility and medium-low temperature deformation properties of recycled asphalt mortar only by the change of ductility, and it needs to be further analyzed and evaluated by rheological tests. Compared to ASTM standards (ASTM D 5, ASTM D 36 and ASTM D 113), the POE/VO composite recycled asphalt performances in penetration degree, softening point, ductility and viscosity are basically meets the requirements.

### 3.2 TeS analysis

**Fig 4:**
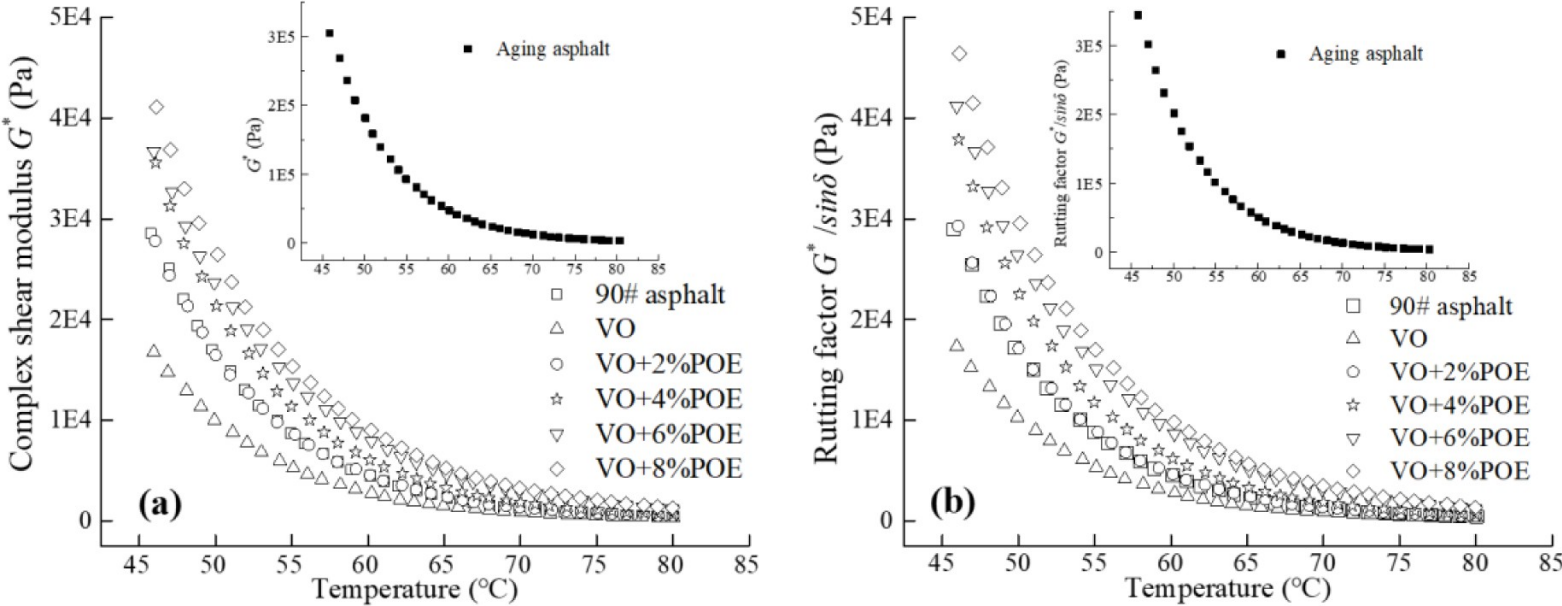
TeS test results of reclaimed asphalt with different VO+POE. (a) G* value; (b) G*/sinδ value.

Fig 4 shows the G* and G*/sinδ of POE/VO recycled asphalt as a function of the test temperature. The graph shows that with the increase of temperature, the G* and G*/sinδ values of the glue gradually decrease, and the higher the temperature, the smaller the difference of G* and G*/sinδ values of the glue. The main reason is that high temperature makes the internal viscous components gradually increase and the elastic components gradually decrease, so that the asphalt mortar gradually transforms from elastic state to viscous state. The order of G* and G*/sinδ values of asphalt mortar is as follows: aged asphalt >VO+ 8%POE > VO+ 6%POE > VO+ 4%POE > matrix asphalt > VO+ 2%POE > VO. The test results show that the addition of VO can partially restore elastic deformation property of asphalt mortar, the main reason is that VO supplements the lightweight components of asphalt, and its linoleic acid composition and aging asphalt produce physical reactions [38]. However, the G*/sinδ of VO asphalt is still lower than it of matrix asphalt, this also demonstrates the necessity of study composite regeneration of aged asphalt with POE and VO, at the same time, the G* and G*/sinδ values of VO recycled asphalt are significantly increased by the addition of POE elastaner, of course, it has been proved that POE can improve the elastic recovery performance and high temperature resistance of the glue, Among them, some VO recycled asphalt of more than 2% POE content can restore the high temperature performance and even exceed the level of matrix asphalt. It is mainly due to the formation of a three-dimensional through network structure formed by the crystalline ethylene chain and the coiled octene chain structure of POE inside the glue, with the increase of POE content, the dense degree of network structure increases. Under the action of external load stress, it can delay the deformation of the mortar of reinforcement support effect and contact friction through the network structure, as a result, the glue system presents more elastomer characteristics [23, 36, 39]. In addition, although the higher G* and G*/sinδ values can improve the deformation resistance of the mortar, but if its value is too high, the brittleness of the mortar will increase, it is easy to crack, and the construction workability of the binder is poor, and the mixing temperature is also too high. Therefore, the recommended content of composite recycled asphalt is VO+4% POE and VO+ 6% POE.

Based on Eq (4), the G^*^/sinδ value and the temperature test results are fitted, the corresponding temperature (critical temperature, *T*_*c*_) of G^*^/sinδ = 1000 Pa is determined, and the specific calculation results are shown in Table 4. It can be found from the table that the *T*_*c*_ value of asphalt after aging is the largest, this also verifies the test result in Section 2.1 that the aged asphalt has the highest softening point. At the same time, the adding of VO to aging asphalt can partially restore its performance, the increase of POE to it can increase the *T*_*c*_ value of the glue, for example, the *T*_*c*_ value of VO+4%POE, VO+6%POE and VO+8%POE respectively increase by 4.1°C, 8.9°C and 10.4°C, compared with those before aging. This indicates that POE can significantly improve the high temperature deformation resistance of VO recycled asphalt.


$$G*/sin\delta =Ae^{BT}$$ (4)

**Table 4:** Critical temperature of asphalt (*T*_*c*_).

Type of Asphalt	Matrix Asphalt	Aged Asphalt	VO	VO+2% POE	VO+4% POE	VO+6% POE	VO+8% POE
*T*_c_(°C)	71.4	89.5	68.4	71.8	75.5	80.3	81.8

In the above formula: G*/sinδ is the rutting factor, T is the test temperature, *A* and *B* are the correlation fitting coefficients.

### 3.3 FrS analysis

**Fig 5:**
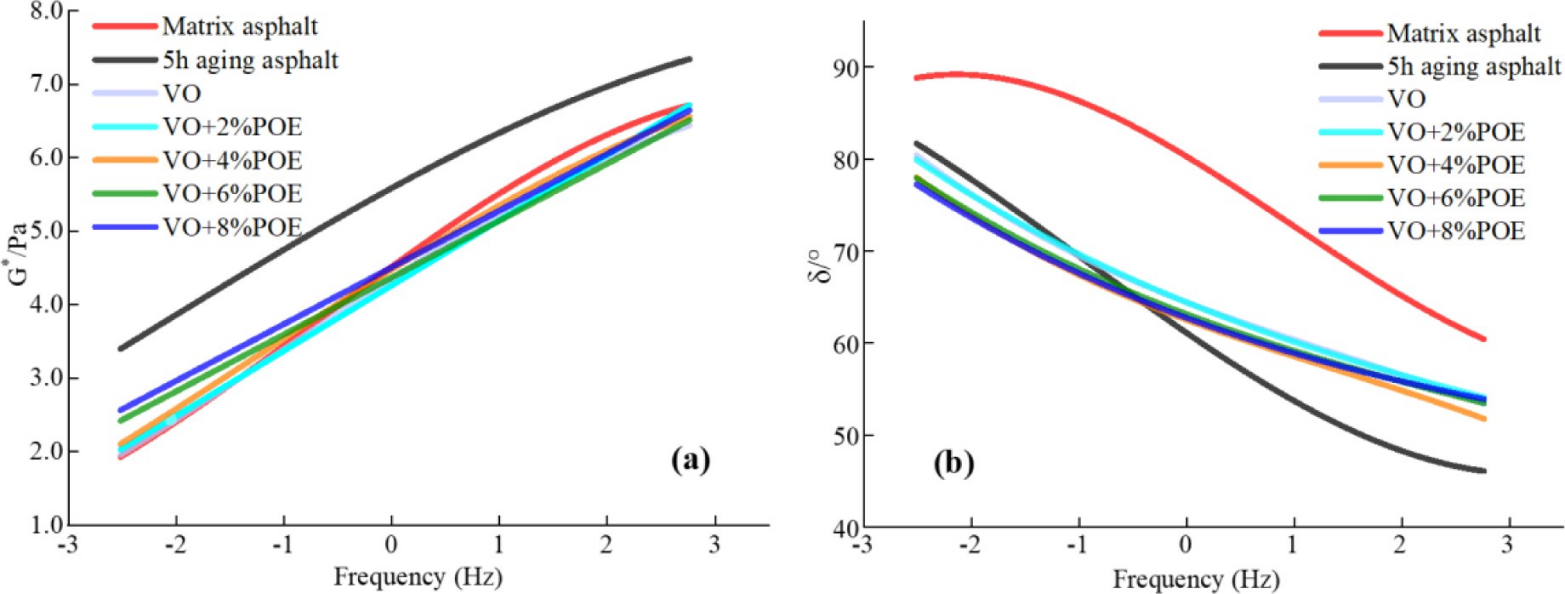
FrS main curves of different recycled asphalt. (a) G* value; (b) *δ* value.

According to the principle of time-temperature equivalence and Williams-Landel- Ferry (WLF) equation, the frequency scan results at each test temperature are translated, superimposed and fitted to obtain the FrS test G* value and *δ* value principal curves for the reference temperature of 40°C (See Fig 5). In the low frequency (high temperature) region, the larger value of G* and the smaller value of *δ*, the stronger of high temperature deformation resistance and elastic recovery performance of asphalt mortar. In the high frequency (low temperature) domain, the smaller value of G* and the smaller value of *δ*, the better of low temperature flexibility.

According to Fig 5, with the increase of load frequency, the G* of asphalt mortar gradually increases and the *δ* value gradually decreases, which is consistent with the above TeS test results, that is, low temperature increases the elastic component and decreases the viscous component in the mortar, resulting in more high temperature deformation resistance of the mortar. This phenomenon is also consistent with the dynamic response law of asphalt pavement in actual use, that is, when the vehicle speed is high and the loading frequency is large, the elastic response of asphalt pavement will be better, as a result, highways tend to have fewer ruts than gas stations and parking lots. Compared with matrix asphalt and recycled asphalt, the G* value of aged asphalt is significantly larger in the high-low frequency domains, while the *δ* value is smaller in the high frequency (low temperature) domain, that is, the viscoelasticity of the aged asphalt is good in the high-low frequency domain, but the creep property is very poor in the high frequency (low temperature) domain. The G* values of recycled asphalt and matrix asphalt in different frequency domain segments are similar, indicating that VO has a significant recycled effect on aging asphalt and effectively reduces the G* value. At the same time, compared with the matrix asphalt, the G* value of POE/VO recycled asphalt is larger in the low frequency (high temperature) domain, and smaller in the high frequency (low temperature) domain, it shows that the high temperature elastic deformation recovery performance and low temperature creep performance of composite recycled asphalt are better than the matrix asphalt. The main reason for it is that the sulfur cross-linking agent improves the interface energy of POE elastoplast and promotes the grafting and cross-linking reactions of the blend system, the side octyl group of POE molecular structure is longer than the side ethyl group, which can produce many bonding points in the mortar and form an interpenetrating network structure, hindering the movement of the asphalt matrix and making the mortar system show more mechanical elasticity [23, 36]. In addition, the δ value of recycled asphalt is lower than it of matrix asphalt in both high and low frequency bands, it shows that the elastic recovery performance of recycled asphalt is better than it of matrix asphalt in the low frequency (high temperature) region, however, its rheological properties in the high frequency (low temperature) domain may not be as good as the matrix asphalt.

### 3.4 MSCR analysis

**Fig 6:**
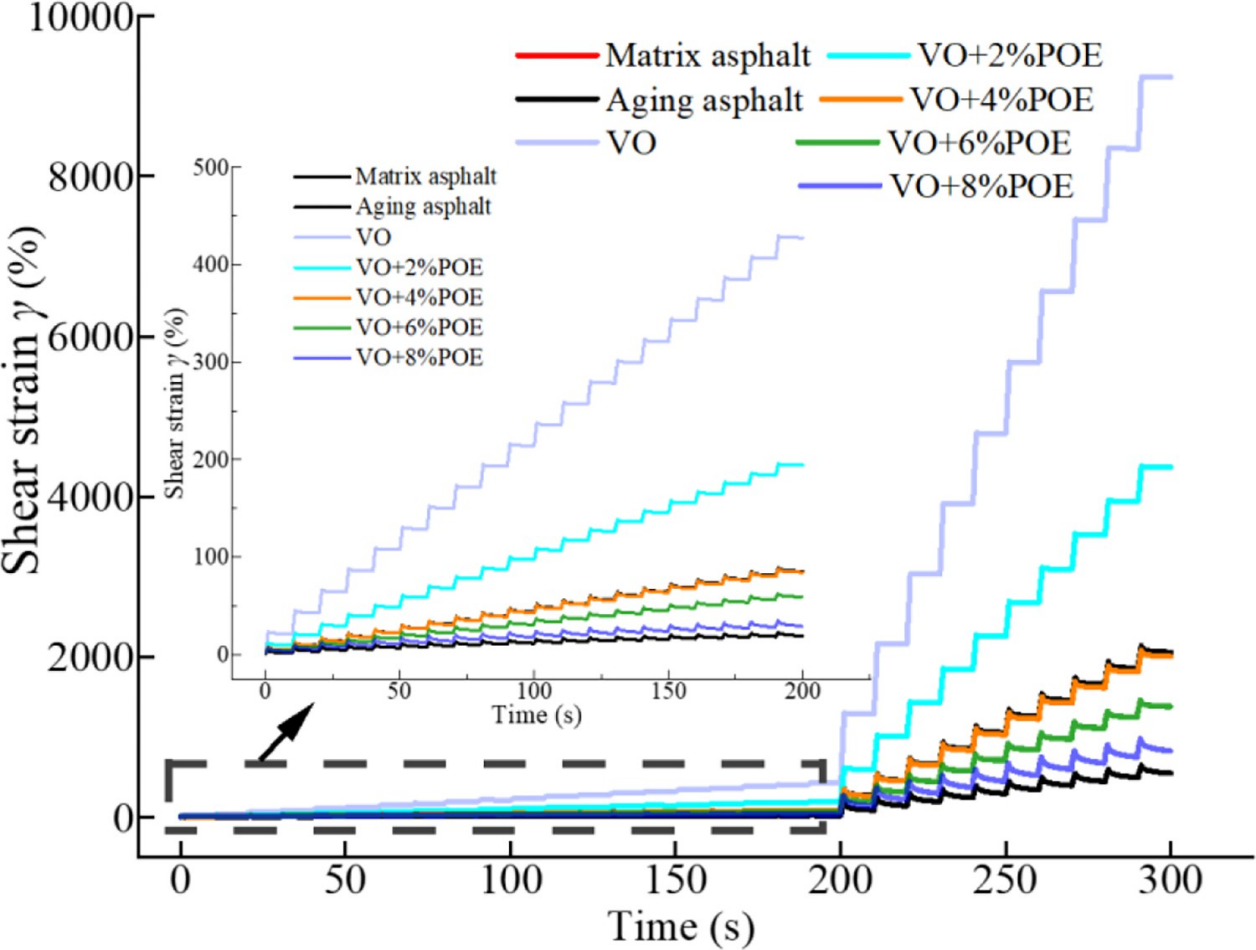
*γ*-*time* curves of different recycled asphalt in MSCR test.

In order to further analyze, the high temperature elastic recovery performance of asphalt mortar, MSCR test was carried out under 58°C test environments for each recycled asphalt mortar, the shear strain-time (*γ*-*time*) curve is shown in Fig 6. It can be seen from the figure that the cumulative strain under the stress level of 0.1kPa is significantly lower than it of 3.2kPa, this verifies that heavy traffic is more likely to cause road ruts and affect the level of road service [40]. The value of asphalt mortar gradually increased with time, indicating that the irrecoverable deformation of each asphalt mortar gradually accumulated with the extension of loading time, the overall order of cumulative strain is as follows, VO>VO+2%POE>matrix asphalt >VO+ 4%POE >VO+ 6%POE >VO+8%POE >aged asphalt, this also verifies the aforementioned DSR test results. The curves of the aged bitumen are significantly lower than those of the other asphalt, it is because the loss of light components in asphalt caused by long-term thermal oxygen aging, and the texture becomes brittle and hard on the macro. The *γ*-*time* curve of VO and VO+2%POE recycled asphalt is significantly higher than it of matrix asphalt, that is, the cumulative shear strain of it is larger, and the resistance to permanent deformation is worse than it of matrix asphalt, this also shows that it is necessary to further improve the high temperature performance of VO recycled asphalt. However, the *γ*-*time* curves of VO+4%POE and matrix asphalt almost coincided, indicating that 8%VO+4%POE content could restore the permanent deformation resistance and elastic recovery ability of aging asphalt to the pre-aging level. The *γ*-*time* curve of VO+6%POE and VO+8%POE recycled asphalt is lower than it of the matrix asphalt, which indicates that its resistance to permanent deformation is better, and it also indicates that POE effectively enhances the deformation resistance and elastic recovery performance of VO recycled asphalt, this may be the reason what POE molecular structure with molecular weight distribution forms an interpenetrating structure with octene chain coiled structure and crystalline ethylene chain as crosslinks, which plays a bonding and buffering role in the glue. At the same time, POE interpenetrates the mesh microstructure to aggregate the mortar components, thereby dispersing and buffering the load impact energy borne by the whole system. The polyolefin elastomers (POE) are thermoplastic elastomers that use metallocene catalysts for in-situ polymerization of ethylene and olefins. The crystalline region of the polyethylene chain (resin phase) acts as a physical crosslinking point and has typical plastic properties. After adding a certain amount of olefins, the crystallization zone of the polyethylene chain is weakened, forming an amorphous region (rubber phase) that exhibits rubber elasticity, possessing the properties of an elastomer. The molecular structure of polyolefin elastomer POE does not have unsaturated double bonds, but a narrow molecular weight distribution and short branched chain structure (with uniform short branched chain distribution). Therefore, it has excellent physical and mechanical properties such as high elasticity, high strength, high elongation, and excellent low-temperature resistance. Therefore, the incorporation of POE improves the elastic deformation recovery performance of the asphalt [23, 37, 40, 41].

**Fig 7:**
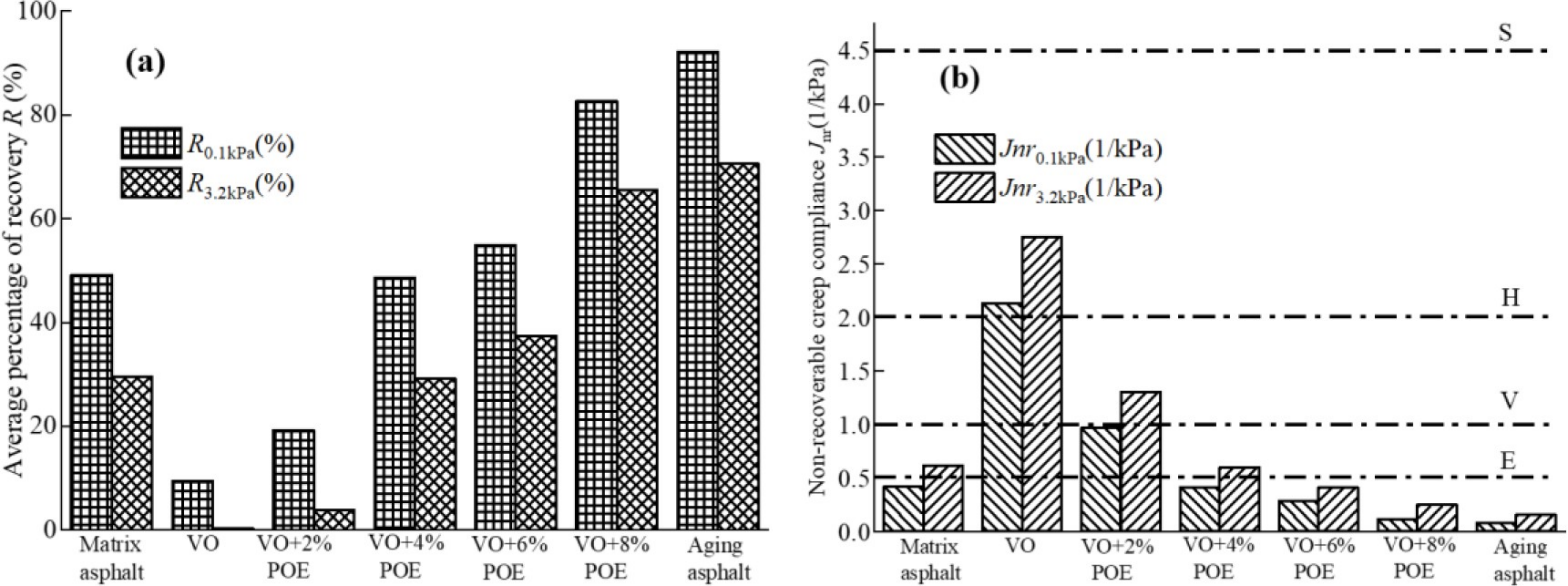
MSCR test results of different recycled asphalt. (a) *R(τ)* value; (b) *Jnr(τ)* value.

Fig 7 shows the test results of *R(τ)* values and *Jnr(τ)* values of different recycled asphalt at different stress levels. The larger value of *R(τ)* and the smaller value of *Jnr(τ)*, the better elastic deformation recovery performance of asphalt mortar, the smaller viscous flow deformation, and the better the high temperature rut resistance performance. It can be seen from the figure that with the increase of stress level, the *R(τ)* value of various types of mucilage decreases while the *Jnr(τ)* value increases, it shows that the increase of stress will increase the irrecoverable deformation of the mortar, this may be caused by damage to the internal structure of the asphalt mortar at high stress levels. Under different stress levels, the *R(τ)* value of aged asphalt is the largest and the *Jnr(τ)* value is the smallest, this indicates that aged asphalt has little irrecoverable deformation, and its performance is close to that of a brittle elastic body, which is also consistent with the aforementioned conclusion that aged asphalt has a higher softening point. The *R(τ)* value of pure VO recycled asphalt is smallest and the *Jnr(τ)* value is the largest, which indicates that pure VO regeneration may cause the problem of insufficient deformation recovery performance of the mortar. In addition, with the increase of POE content, the *R(τ)* and *Jnr(τ)* values of composite recycled asphalt are gradually close to and better than the pre-aging level, taking the 0.1kPa stress level as an example, the *R*_*0*.*1kPa*_ increases by -60.88% (VO+2%POE), -1.14% (VO+4%POE), 11.62% (VO+6%POE) and 68.12% (VO+8%POE), and its *Jnr*_*0*.*1kPa*_ respectively decreased -130.82% (VO+2%POE), 1.12% (VO+4%POE), 31.78% (VO+6%POE), and 72.58% (VO+8%POE). It shows that the addition of POE to recycled asphalt can improve the deformation resistance of the mortar under repeated load and reduce the viscous flow deformation, this is the reason of part of the asphalt matrix is adhered to the network structure formed by POE cross-linking as structural asphalt, the locking and entanglement effect between the two and the effective interface bonding promote the condensation and dispersion of the impact stress of the mortar system components. Thus, the deformation characteristics of the system are improved [23, 36, 37, 39].

Referring to the classification requirements of the standard (AASHTO M 332–14) for the adaptability of asphalt mortar to the traffic environment, *Jnr*_*3*.*2kPa*_ and *Jnr*_*diff*_ were used as the classification indexes [28] to classify the adaptability of different recycled asphalt mortar to the environment, the specific results are shown in Table 5. According to Table 5, the recycled asphalt of 8%VO+2%POE content is suitable for 58°C heavy load traffic and below, and the recycled asphalt of 8%VO+4%POE content is suitable for 58°C super heavy traffic and below, the recycled asphalt of 8%VO+6%POE and 8%VO+8%POE content is suitable for 58°C extremely heavy traffic pavement and bridge deck pavement layer.

**Table 5:** Traffic grade of asphalt.

Asphalt type	Matrix Asphalt	Aged Asphalt	VO	VO+2%POE	VO+4%POE	VO+6%POE	VO+8%POE
*Jnr*_*3*.*2kPa*_ (1/kPa)	0.6166	0.1562	2.7532	1.3042	0.6006	0.4112	0.2481
*Jnr*_*-diff*_ (%)	47.23	43.4	28.99	34.91	45.04	43.92	46.07
Traffic grade	V	E	S	H	V	E	E

In Table 5, S denotes standard designation, H denotes high designation, V denotes very high designation and E denotes extremely high designation.

### 3.5 BBR analysis

**Fig 8:**
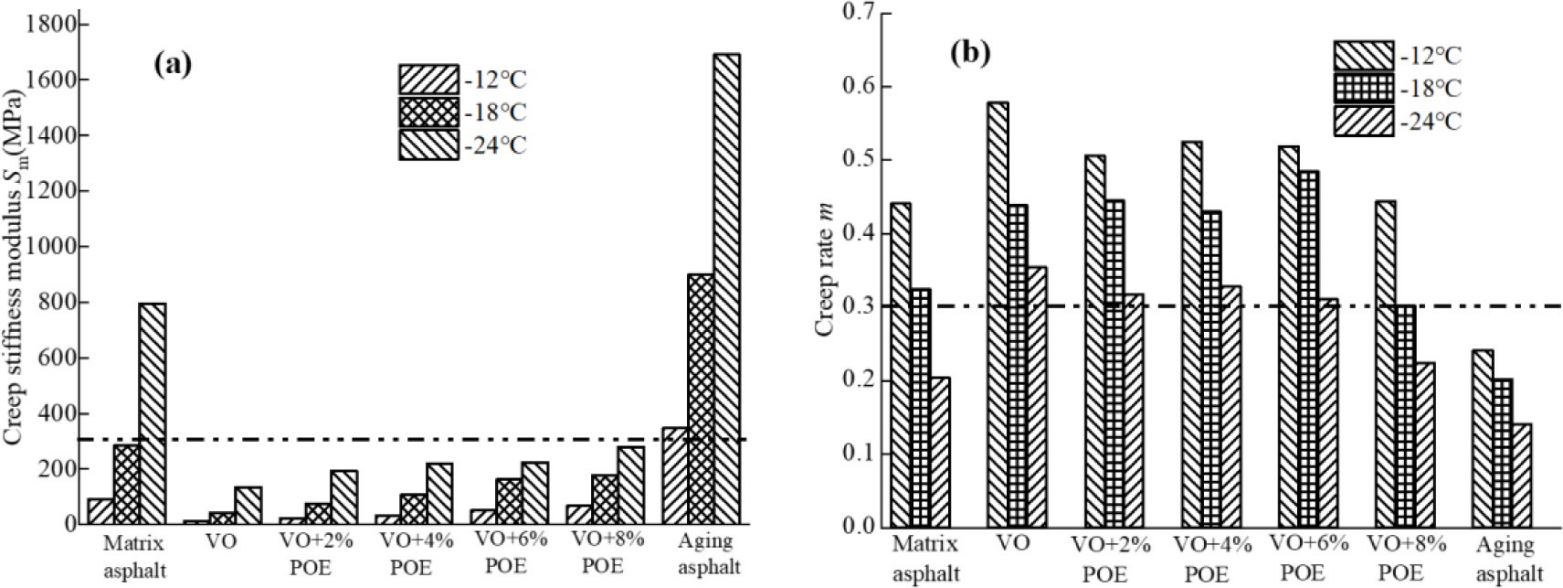
BBR test results of different recycled asphalt. (a) *S*_*m*_ value; (b) *m* value.

Fig 8 shows the variation of *S*_*m*_ and m values of the BBR test results of recycled asphalt at -12°C, -18°C and -24°C test temperatures. It can be seen from the figure that with the increase of test temperature, the *S*_*m*_ value of asphalt mortar gradually decreases while the value gradually increases, that is, the content of viscous components in the mortar increases and the fluidity is enhanced with the increase of temperature. Compared with the matrix asphalt and recycled asphalt, the *S*_*m*_ value of the aged asphalt is larger and the *m* value is smaller, which exceeds the standard requirements (i.e. *S*_*m-max*_ = 300, *m*_*min*_ = 0.3) [42, 43], it shows that its low temperature deformation ability is poor, which is also consistent with the conclusion that the aging asphalt has a large modulus and is easy to appear low temperature brittle fracture in TeS test results. The addition of VO can significantly reduce the *S*_*m*_ value and increase the *m* value of aging asphalt, that is, improve its low-temperature stress relaxation performance, this is mainly because VO can supplement the light components in the aged asphalt, and its linoleic acid component has a physical reaction with the aged asphalt to improve the low temperature creep ability of the mortar [38]. However, with the increase of POE content, the low-temperature properties of POE/VO recycled asphalt are developing in an unfavorable direction, resulting in a decrease in the delay and low-temperature stress relaxation properties of the mortar, and more brittleness characteristics. Taking -24°C as an example, compared with pure VO recycled asphalt, its *S*_*m*_ value respectively increases by 43.70% (VO+2%POE), 62.22% (VO+4%POE), 65.93% (VO+6%POE) and 107.41% (VO+8%POE), and their *m* values are reduced by 10.45% (VO+2%POE), 7.34% (VO+4%POE), 12.15% (VO+6%POE) and 32.72% (VO+8%POE). This is because POE forms an interpenetrating network structure with locking and winding effects in the mortar, thus limiting the mobility of the asphalt matrix, in addition, the extension flexibility of POE is reduced at low temperature, resulting in the brittle hardness of the mortar system at low temperature. However, the *S*_*m*_ and *m* values of POE/VO recycled asphalt are relatively concentrated, and the low temperature performance of composite recycled asphalt with 0%~6%POE content meets the requirements of the above standards at various temperatures.

## 4. Conclusions

In this paper, the following main research conclusions are obtained:

(1) The addition of VO to aging asphalt has a good regeneration effect, which can significantly improve its conventional physical properties, with the increase of VO content, the penetration and ductility of recycled asphalt increased, while the softening point and viscosity decreased. The optimum content of VO in recycled asphalt is 8%, but its high temperature deformation resistance is still lower than the matrix asphalt.

(2) The TeS analysis shows that the G* and G*/sinδ values of VO recycled asphalt can be significantly increased by adding POE, when the content is more than 2%, the high temperature performance of the asphalt can be recovered and even exceed the level of the matrix asphalt, and the critical temperature value can be increased by 4~10°C. In view of the fact that high G*/sinδ is easy to cause brittle fracture of the mortar, the recommended POE content based on high temperature performance is 4% and 6%.

(3) FrS analysis shows that the elastic deformation performance of POE/VO recycled asphalt in low frequency (high temperature) domain and creep recovery performance in high frequency (low temperature) domain are close to or even better than matrix asphalt.

(4) MSCR analysis shows that under different stress levels (0.1kPa and 3.2kPa), the *R(τ)* and *Jnr(τ)* values of POE/VO composite recycled asphalt are gradually close to and better than matrix asphalt with POE content. At the same time, POE/VO recycled asphalt with more than 4%POE content is suitable for the pavement and bridge deck pavement of 58°C overweight or extremely heavy traffic.

(5) The increase of POE content is not good for the low-temperature performance of POE/VO recycled asphalt, which will lead to the decrease of the ductility and low-temperature stress relaxation. However, the values of *S*_*m*_ and *m* are relatively concentrated, and the low temperature performance of 0%~6%POE composite recycled asphalt meets the requirements of AASHTO standard. Considering various physical properties and rheological properties, the optimal dosage of 8%VO+4%POE and 8%VO+ 6%POE is recommended.

## Supporting information

S1 Data (XLS)

## References

[1] Ji J.; Yao H.; Suo Z.; You Z.P.; Li H.X.; Xu S.F.; et al. Effectiveness of Vegetable Oils as Rejuvenators for Aged Asphalt Binders. *Journal of Materials in Civil Engineering* 2017, 29(3), D4016003.

[2] Suo Z.; Chen H.; Zhang A.; Nie L. UV Aging Mechanism and Road Performance of Waste Vegetable Oil Recycled Asphalt. *Materials Reports* 2021, 35(Z1), 662–668.

[3] Micaelo R.; Al-Mansoori T.; Garcia A. Study of the mechanical properties and self-healing ability of asphalt mixture containing calcium-alginate capsules. *Construction and Building Materials* 2016, 123, 734–744.

[4] Zuo F.; Ye F.; Song Q.Q. Influence of RAP Content on Road Performance of Recycled Asphalt Mixture. *Journal of Jilin University (Engineering and Technology Edition)* 2020, 50(4), 1403–1410.

[5] Lv S. T; Liu, C. C.; Yao, H.; Zheng, J. L. Comparisons of Synchronous Measurement Methods on Various Moduli of Asphalt Mixtures. *Construction and Building Materials* 2018, 158, 1035–1045.

[6] Hong J.; Zhang H.L.; Kang H.N.; Liu T.; Wang N. Research Progress on Vegetable Oil Rejuvenator of Asphalt. *Applied Chemical Industry* 2020, 49(6), 1477–1480+1484.

[7] Zadshir M.; Oldham D.; Hosseinnezhad S.; Fini E. Investigating Bio-rejuvenation Mechanisms in Asphalt Binder Via Laboratory Experiments and Molecular Dynamics Simulation.*Construction and Building Materials* 2018, 190, 392–402. doi: 10.1016/j.conbuildmat.2018.09.137

[8] Luo Y.F.; Zhang K. Review on Performance of Asphalt and Asphalt Mixture with Waste Cooking Oil.*Materials* 2023, 16(4), 1341. doi: 10.3390/ma16041341 36836971 PMC9965389

[9] Chen A.Q.; Hu Z.A.; Li M.L.; Bai T.; Xie G.J.; Zhang Y.X.; et al. Investigation on the Mechanism and Performance of Asphalt and Its Mixture Regenerated by Waste Engine Oil.*Construction and Building Materials* 2021, 313, 125411.

[10] Xu Y.; You Z. P.; Dai Q.L. Performance Evaluation of Asphalt Binder Modified by Bio-oil Generated from Waste Wood Resources. *International Journal of Pavement Research and Technology* 2013, 6(4),431–439.

[11] Li X.M.; Wei D.B.; Yao Z.J.; Li B. Effects of rejuvenators on rheological properties and microstructures of aged asphalt. *Journal of Building Materials* 2018, 21(06),992–999.

[12] Yan Z.H.; Zhang Q.Z. Study on Performance of Bio-oil Regenerator Rejuvenated SBS Aged Asphalt Binder and Hot Recycled Mixture. *New Building Materials* 2020, 47(03),83–87.

[13] Tang B.M.; Cao X.X.; Zhu H.Z.; Cao X.J. Pavement Properties of Bio-oil Rejuvenated Asphalt Binder. *China J*. *Highw*. *Transp*. 2019,32(04), 207–214.

[14] Liu Y. Y.; Qiu Q. B.; Ji W. Q.; Pang L. L.; Li L.; Tang W.; et al. Effect of RAP Classification on Road Performance Variability of Hot Recycled Asphalt Mixture. *Highway Engineering* 2021,46(1), 68–72+ 111.

[15] Suo Z.; Chen H.; Yan Q.; Tan Y.T.; Li X.H.; Zhang A. Laboratory Performance Evaluation on the Recovering of Aged Bitumen With Vegetable Oil Rejuvenator. *Frontiers in Materials* 2021, 8,650809.

[16] Elkashef M.; Williams R.C.; Cochran E.W. Thermal and Cold Flow Properties of Bio-derived Rejuvenators and Their Impact on the Properties of Rejuvenated Asphalt Binders. *Thermochimica Acta* 2019, 671(1),48–53.

[17] Cao Z.L.; Chen M.Z.; Liu Z.Y.; He B.Y.; Yu J.Y.; Xue L.H. Effect of Different Rejuvenators on the Rheological Properties of Aged SBS Modified Bitumen in Long Term Aging. *Construction and Building Materials* 2019, 215,709–717.

[18] Park K.B.; Kim J.S.; Pahlavan F.; Fini E.H. Biomass Waste to Produce Phenolic Compounds as Antiaging Additives for Asphalt. *Acs Sustainable Chemistry &* *Engineering* 2022, 10(12),3892–3908.

[19] Gong M.H.; Yang J.; Zhang J.Y.; Zhu H.R.; Tong T.Z. Physical–chemical Properties of Aged Asphalt Rejuvenated by Bio-oil Derived from Biodiesel Residue. *Construction and Building Materials* 2016, 105,35–45.

[20] Zhang D.; Chen M.; Wu S.; Zheng J.; Sang Y. Low Temperature Properties of Waste Edible Vegetable Oil Rejuvenated Asphalt Binder with Recycled Tire Rubber. *Journal of Testing and Evaluation* 2018, 46(2),602–609.

[21] Li B.; Liu W.Y.; Nan X.L.; Yang J.; Tu C.Z.; Zhou L. Development of Rejuvenator Using Waste Vegetable Oil and Its Influence on Pavement Performance of Asphalt Binder under Ultraviolet Aging. *Case Studies in Construction Materials* 2023, 18,e01964.

[22] Gokalp I.; Uz V.E. Utilizing of Waste Vegetable Cooking Oil in Bitumen: Zero Tolerance Aging Approach. *Construction and Building Materials* 2019, 227,116695.

[23] Wang S. F.; Xie Y. G. Crumb Tire Rubber Polyolefin Elastomer Modified Asphalt with Hot Storage Stability. *Progress in Rubber Plastics and Recycling Technology* 2016, 32(1),25–38.

[24] Xie Y.G.; Yao H.R.; Huang J. Structure and Properties of POE Modified Asphalt. *China Elastomerics* 2013,23(03),29–34.

[25] Ran L.; Guo X.T.; Yan Y.R. Study on Properties of Ethylene-Octene Grafted Polymer Modified Asphalt. *China Petrochemical Technology* 2020,27(07),6–9.

[26] Gu Z. F.; Zhao X. W.; Zhang Z.; Luo Y.F. Aging Resistance of Asphalt Mixture Modified by Polyolefin Elastomer/Styrene-butadiene-styrene Block Copolymer. *China Synthetic Rubber Industry* 2022,45(5), 386–392.

[27] Zeng G. P.; Shen A. Q.; Lyu Z. H.; Kang C.; Cui H.X.; Ren G.P.; et al. Research on anti-aging properties of POE/SBS compound-modified asphalt in high-altitude regions. *Construction and Building Materials* 2023, 376, 131060.

[28] Ye Z.; Zhao Y.C. Polyolefin Elastomer Modified Asphalt: Performance Characterization and Modification Mechanism. *Buildings* 2023, 13(5), 1291.

[29] ASTM D2872-12e1. Standard Test Method for Effect of Heat and Air on a Moving Film of Asphalt (Rolling Thin-Film Oven Test); ASTM International: West Conshohocken, PA, USA, 2012.

[30] ASTM D5/D5M-13. Standard Test Method for Penetration of Bituminous Materials; ASTM International: West Conshohocken, PA, USA, 2013.

[31] ASTM D36/D36M-14e1. Standard Test Method for Softening Point of Bitumen (Ring-and-Ball Apparatus); ASTM International: West Conshohocken, PA, USA, 2014.

[32] ASTM D113-17. Standard Test Method for Ductility of Asphalt Materials; ASTM International: West Conshohocken, PA, USA, 2017.

[33] ASTM D4402/D4402M-15. Standard Test Method for Viscosity Determination of Asphalt at Elevated Temperatures Using a Rotational Viscometer; ASTM International: West Conshohocken, PA, USA, 2015.

[34] AASHTO T 315–2019. Standard Method of Test for Determining the Rheological Properties of Asphalt Binder Using a Dynamic Shear Rheometer (DSR); AASHTO International: Washington, DC, USA, 2019.

[35] ASTM D 6648–08. Standard Test Mesthod for Determining the Flexural Creep Stiffness of Asphalt Binder Using the Bending Beam Rheometer (BBR); ASTM International: West Conshohocken, PA, USA, 2008.

[36] Elkashef M.; Williams R. C.; Cochran E. Effect of Asphalt Binder Grade and Source on the Extent of Rheological Changes in Rejuvenated Binders. *Journal of Materials in Civil Engineering* 2018, 30(12),04018319.

[37] Xie Y.G.; Wang S.F.; Zhang Y. Study on Properties of POE/Rubber Powder Compound Modified Asphalt. *China Sino-foreign Highway* 2013, 33(06), 298–302.

[38] Suo Z.; Ji J.; Man Q.; Xu S.F.; Sun L.J. Performance Evaluation of Regenerated Asphalt With Vegetable Oil. *Journal of Beijing University of Technology* 2016,42(07),1062–1065.

[39] Wang P.; Dong Z.J.; Tan Y.Q.; Liu Z.Y. Anti-ageing Properties of Styrene-butadiene- styrene copolymer-modified Asphalt Combined with Multi-walled Carbon Nanotubes. *Road Materials and Pavement Design* 2017, 18(3), 533–549.

[40] Zhang Z.; Zhang H.L.; Gao Y.; Kang H.N. Laboratory Evaluation of the Effect of Kapok Fibers on the Rheological and Fatigue Properties of Bitumen. *Construction and Building Materials* 2021, 272,121819.

[41] Mouillet V.; Fabienne F.; Besson S. Ageing by UV Radiation of An Elastomer Modified Bitumen. *Fuel* 2008,87(12), 2408–2419.

[42] AASHTO MP 19–10. Standard Specification for Performance-Graded Asphalt Binder Using Multiple Stress Creep Recovery (MSCR) Test. *American Assciation of State Highway and Transportation Officials* 2010.

[43] Cui P. Study on Rheological Properties of Composite Asphalt Modified with CaCO_3_/Nano-SiO_2_/SBS. *Journal of China & Foreign Highway* 2021, 41(5),292–295.30.

